# Somatosensory cross-modal activation and changes in cortical somatosensory evoked potential responses in single-sided deafness: an EEG study

**DOI:** 10.3389/fnins.2025.1618134

**Published:** 2025-09-05

**Authors:** Ghislain Sofack, Kotaiba Raouafi, Sven P. Heinrich, Antje Aschendorff, Thomas Wesarg, Susan Arndt, Pascale Sandmann, Iva Speck

**Affiliations:** ^1^Department of Otorhinolaryngology-Head and Neck Surgery, Medical Center – University of Freiburg, Freiburg, Germany; ^2^Department of Nuclear Medicine, Medical Center – University of Freiburg, Freiburg, Germany; ^3^Eye Center, Medical Center – University of Freiburg, Freiburg, Germany; ^4^Department of Otorhinolaryngology-Head and Neck Surgery, University of Oldenburg, Oldenburg, Germany

**Keywords:** single-sided deafness (SSD), cortical somatosensory evoked potentials (CSEP), cross-modal plasticity, sLORETA, somatosensory stimulation, neuroplasticity

## Abstract

**Background:**

The neural mechanisms underlying somatosensory processing in individuals with acquired single-sided deafness (SSD) and potential central neuronal cross-modal reorganization remain largely unexplored. This study investigates the impact of SSD on somatosensory perception and attentional processing.

**Methods:**

Electrophysiological responses using EEG, and behavioral measures (discrimination thresholds, hit rates and reaction times) were assessed in adults with acquired SSD and normal-hearing (NH) controls for vibrotactile stimulation at two distinct frequencies. Differences in cortical somatosensory evoked potentials between adults with acquired SSD and normal-hearing (NH) controls, focusing on peak amplitudes and peak times of key event-related potential components (P50, N70, P100, N140, and P3b) and their cortical generators were assessed.

**Results:**

While both groups exhibited comparable behavioral performance, significant differences emerged in electrophysiological responses. Individuals with SSD showed increased P3b amplitude (albeit non-significant) and significantly delayed P3b peak times, indicating that individuals with acquired SSD exhibit alterations in attentional mechanisms associated with somatosensory perception. In addition, source localization analysis of the P50 component using standardized low-resolution brain electromagnetic tomography (sLORETA) revealed group differences in cortical activation patterns, with SSD individual showing additional recruitment of auditory-related areas, including the superior temporal gyrus, the middle temporal gyrus and the insula. This further supports the notion of compensatory neuroplasticity in auditory pathways following severe to profound unilateral hearing loss.

**Conclusion:**

Our results indicate that SSD is associated with neural reorganization in somatosensory and auditory pathways. The observed modifications in both early and late somatosensory responses, coupled with alterations in source activity, suggest that individuals with SSD engage alternative neural mechanisms when processing vibrotactile stimuli, differing from the typical patterns observed in NH individuals. Understanding these changes prior to cochlear implantation will facilitate the development of personalized auditory rehabilitation strategies following cochlear implantation.

## 1 Introduction

A form of hearing loss that has long been neglected is single-sided deafness (SSD), which is defined as profound hearing loss in one ear with normal or nearly-normal hearing in the other ear. Monaural hearing impairs speech understanding in background noise (Hol et al., 2010; Arndt et al., 2011, 2017a; Jakob et al., 2021) and localization of sound sources (Feuerstein, 1992; Hol et al., 2010; Wie et al., 2010; Jakob et al., 2021). SSD leads to significantly poorer hearing in the better-hearing ear compared to an age-correlated normal-hearing (NH) control group (Arndt et al., 2020; Speck et al., 2024). Additionally, it can cause exhaustion, frustration, and social isolation as a result of heightened stress levels and increased listening effort (Andersson et al., 2009; Hol et al., 2010; Kitterick et al., 2014; Alhanbali et al., 2017).

Literature shows that SSD induces structural and functional changes of the brain; for instance, the auditory cortex, higher-order cognitive areas and the dorsal attention network (Fan et al., 2015; Wang et al., 2016; Shang et al., 2018; Speck et al., 2020; Weglage et al., 2022). Various studies on individuals with different degrees of hearing loss (Levänen and Hamdorf, 2001; Campbell and Sharma, 2016; Sharma and Glick, 2016; Zhang et al., 2016; Glick and Sharma, 2017; Kral and Sharma, 2023) have revealed that, in adults with acquired SSD, neural changes extend beyond auditory processing and involve other sensory modalities (e.g., somatosensation). Specifically, vibrotactile stimulation in adults with prelingual bilateral deafness (Levänen et al., 1998), in adults with age-related hearing loss (ARHL) (Cardon and Sharma, 2018) and in a case-study on a child with SSD (Sharma et al., 2016), resulted in activation of the central auditory system. Furthermore, Levänen and Hamdorf (2001) demonstrated that deafness not only influences the behavioral reactions to auditory stimuli (e. g. localization of sound sources) (Arndt et al., 2017b) and neuronal computation of auditory stimuli (e.g., cortical auditory evoked potential to the syllable /ba/) (Sharma et al., 2016) but also impacts vibrotactile sensation. Adults with prelingual bilateral deafness exhibit better discrimination of suprathreshold vibrotactile changes than adults with NH (Levänen and Hamdorf, 2001). Despite these insights the extent to which cross-modal plasticity influences somatosensory processing in acquired SSD remains unclear.

Somatosensory stimulus processing is predominantly contralateral, meaning that sensory stimuli applied to one side of the body are primarily processed in the opposite hemisphere of the brain. This organization is well-established in the primary somatosensory cortex (S1), where afferent inputs from peripheral mechanoreceptors project to the contralateral cortical regions via the dorsal column-medial lemniscus pathway.

Using the electroencephalogram (EEG) we can record event-related potentials (ERPs) – electrical potential that are time-locked to specific sensory or cognitive events (Sur and Sinha, 2009; Ghani et al., 2020). The high temporal resolution of ERPs enables us to track distinct steps of cortical processing. Cortical somatosensory evoked potentials (CSEPs) reflect the brain response to somatosensory stimuli. These responses include early exogenous components such as P50, N70, P100, and N140, which are associated with initial sensory encoding in the somatosensory pathway (Hämäläinen et al., 1990; Michel and Murray, 2012; Staines et al., 2014; Biasiucci et al., 2019), as well as later endogenous components like the P300, which are thought to reflect higher-order cognitive processes such as attention and stimulus evaluation (Polich, 2007; Sur and Sinha, 2009). In NH controls, P50 is generated in the S1, reflecting early exogenous sensory encoding of tactile input. The P100 and N140 components originate from the secondary somatosensory cortex (S2) (Hämäläinen et al., 1990; Staines et al., 2014), and are associated with higher-order processing, including somatosensory discrimination and integration.

The P300 component, a late positive endogenous potential (300–800 ms post-stimulus) that is elicited in the context of an oddball task, reflects higher-order cognitive processing, as it has been associated with attention allocation and decision making (Ritter and Vaughan, 1969; Polich, 2007; Sur and Sinha, 2009). The P300 is generally subdivided into two functionally and topographically distinct main subcomponents: P3a and P3b. The P3a is associated with the involuntary orienting of attention toward novel or unexpected stimuli and is most prominent over frontal-central scalp regions. In contrast, the P3b is elicited in active oddball paradigms; experimental designs in which infrequent, task-relevant target stimuli are interspersed among frequent, non-target standard stimuli, and reflects attentional resource allocation, working memory updating, and the conscious evaluation of significant stimuli (Polich, 2007; Verleger, 2020). This component has been extensively studied in auditory, visual and vibrotactile modalities in participants with bilateral hearing loss and those with cochlear implants (CIs) (Micco et al., 1995; Hauthal et al., 2015; Tao et al., 2022; Foo et al., 2024), those with prelingual profound hearing loss (González-Garrido et al., 2017) and also in participants with SSD having a CI (Wedekind et al., 2021; Voola et al., 2023). For example, Hauthal et al. (2015) found significantly larger tactile P3b amplitudes in adults with bilateral deafness compared to adults with NH, during a visuo-tactile stimulation task. In addition, Tao et al. (2022) found that P3b amplitudes were significantly larger and latencies were significantly shorter for the NH than for the CI group in an auditory oddball paradigm. In SSD-CI users, Wedekind et al. (2021) observed no significant difference in P3b responses between the NH and CI ears, but they found that the P3b amplitude correlated with masked speech reception thresholds, indicating its potential as a marker of spectro-temporal processing efficiency. Similarly, Voola et al. (2023) using a semantic oddball paradigm in the presence of background noise demonstrated that P3b measures can assess how SSD individuals integrate degraded CI input with normal acoustic hearing. These studies collectively support the notion that alterations in P300 responses across sensory modalities can reflect compensatory neuroplasticity and reorganization of attentional control mechanisms in response to sensory deprivation. While substantial research has examined the P3b in auditory and visual modalities in participants with SSD, studies employing somatosensory stimuli in this population remain scarce. Therefore, investigating the somatosensory P3b component in the present study offers novel and critical insights into how SSD may influence both early sensory encoding and the recruitment of cognitive resources during somatosensory perception.

The present study aimed to investigate how partial auditory deprivation, as seen in individuals with acquired SSD, affects somatosensory processing. To this, we employed EEG to examine changes in the early exogenous and late endogenous CSEPs during vibrotactile stimulation using a vibrotactile oddball paradigm. In addition, we recorded reaction time (RT) and accuracy to vibrotactile stimulation in adults with acquired SSD to identify possible behavioral changes compared to NH controls.

Firstly, we specifically examined how SSD affects early sensory processing, focusing on exogenous CSEPs such as the P50 component. We hypothesized that alterations in this component, may reflect changes in early-stage cortical processing of somatosensory information, potentially recruiting additional cortical areas. Previous studies investigating bilaterally deaf subjects have demonstrated that neural activation patterns influence speech perception following cochlear implantation (Giraud and Truy, 2002; Lee et al., 2003, 2007; Jeong Lee et al., 2005; Kim et al., 2016) including cross-modal activation of the auditory cortex during vibrotactile stimulation (Kral and Sharma, 2023). Similarly, in subjects with SSD, changes in neural activation patterns (Speck et al., 2020; Karoui et al., 2023; Peter et al., 2024) and a correlation between neural activation and CI outcome (Speck et al., 2022) have been revealed. Building on this, we aimed to investigate whether adults with acquired SSD show cross-modal activation of the auditory cortex during vibrotactile stimulation. This might allow us to establish a biomarker for a better prediction of CI outcome in adults with acquired SSD and therefore to optimize the indication for CI, and therapy and rehabilitation of CI users. To assess these changes, we performed source localization analysis using standardized low-resolution brain electromagnetic tomography (sLORETA) (Sadat-Nejad and Beheshti, 2021) to identify the origin of the early exogenous CSEPs in adults with acquired SSD and in age- and sex-matched NH controls. In the present study, right index finger stimulation was used to elicit cortical somatosensory responses, which, under typical conditions, is expected to be predominantly localized in the contralateral (left) somatosensory cortex. However, in cases of sensory deprivation, such as SSD, neural reorganization may alter this typical pattern, potentially leading to recruitment of additional bilateral cortical areas beyond the expected somatosensory regions.

Secondly, we aimed to investigate behavioral changes in adults with acquired SSD that might result in changes of later endogenous ERPs (P3b). We hypothesized that adults with acquired SSD show faster and more accurate discrimination of vibrotactile changes compared to an age- and sex-matched NH control group, and that these behavioral changes would also be reflected in neural processing, as indicated by changes in the amplitudes and peak times of P3b components.

Finally, we compare electrical brain activity between adults with acquired SSD with the poorer-hearing ear on the right (RSSD) and the left (LSSD). Based on the theory of right ear advantage (REA) we expect that the loss of the “dominant” right ear in adults with acquired RSSD will lead to greater changes as the right ear provides faster auditory processing to the left, language-dominant hemisphere (Prete et al., 2018). We therefore hypothesized that adults with acquired RSSD discriminate vibrotactile changes faster and more accurately, resulting in changes of later endogenous ERPs (P3b) than adults with acquired LSSD. Additionally, we expect greater cross-modal activation of the auditory pathway in adults with acquired RSSD compared to LSSD.

## 2 Materials and methods

### 2.1 Participants

We prospectively enrolled adult participants. Relevant neurological or psychiatric diseases (e.g., cognitive impairment, epilepsy, cerebrovascular disease, brain tumor) led to exclusion. All participants were right-handed, as determined by the Edinburg Handedness Inventory (Oldfield, 1971). The individual patient characteristics are reported in the Supplementary Table 1.

Twenty individuals with acquired SSD (RSSD: *n* = 11, LSSD: *n* = 9) were included in the present study (Table 1) after screening 96 participants that presented to the Department of Otorhinolaryngology Freiburg. SSD was defined according to the consensus paper by Vincent et al. (2015). This required an unaided hearing threshold of ≥70 dB HL in the poorer-hearing ear and an unaided hearing threshold ≤30 dB HL up to 4 KHz in the better-hearing ear. We saw no significant difference between RSSD and LSSD in age, hearing loss in the frequencies 500, 1,000, 2000, and 4,000 Hz (four frequency pure-tone average, 4PTA) for the better- and poorer-hearing ear, age at onset of deafness as well as duration of deafness (Table 1). All individuals with acquired SSD underwent the Hopkins Verbal Learning-test to screen for cognitive impairment (Belkonen, 2011). No included individuals with acquired SSD showed signs of cognitive impairment.

**Table 1:** Characteristics of included individuals with SSD and NH controls.

Parameter	SSD (all)	SSD with right poorer-hearing ear (RSSD)	SSD with left poorer hearing ear (LSSD)	Wilcoxon Signed Rank or Chi-squared test	NH controls
*n*	20	11	9		20
Age	50 ± 10 [32; 66]	49 ± 10 [36; 66]	52 ± 11 [32; 66]	*p* = 0.46.	52 ± 11 [31; 65]
Sex	*F*, *n* = 8 *M*, *n* = 12	*F*, *n* = 4 *M*, *n* = 7	*F*, *n* = 4 *M*, *n* = 5	*p* = 1	*F*, *n* = 9 *M*, *n* = 11
Air-conduction 4PTA [dB HL]					
Better-hearing ear	9 ± 8 [0.75; 35]	10 ± 10 [0.75; 35]	8 ± 5 [2; 21]	*p* = 0.62.	10 ± 6 [2; 22]
Poorer-hearing ear	102 ± 20 [70; 130]	103 ± 21 [71; 130]	102 ± 21 [70; 130]	*p* = 0.94	11 ± 7 [2; 24]
Clinical parameters					
Age at onset of deafness [years]	42 ± 18 [6; 63]	38 ± 19 [6; 63]	47 ± 16 [11; 61]	*p* = 0.26	N/A
Duration of deafness [years]	8 ± 13 [0.2; 41]	11 ± 15 [0.5; 41]	5 ± 9 [0.2; 27]	*p* = 0.23	N/A

Mean ± standard deviation [range]; *p < 0.05, **p < 0.01, ***p < 0.001 for comparisons between SSD subgroups; dB, decibel; NH, normal hearing; 4PTA, pure-tone average of 0.5, 1, 2 and 4 kHz; SSD, single-sided deafness.

For the control group we enrolled 20 age- and sex-matched individuals with NH (Table 1). NH was objectified by a 4PTA ≤ 20 dB hearing loss. Individuals with NH did not undergo screening for cognitive impairment.

### 2.2 Stimuli and experimental task

**Figure 1:**
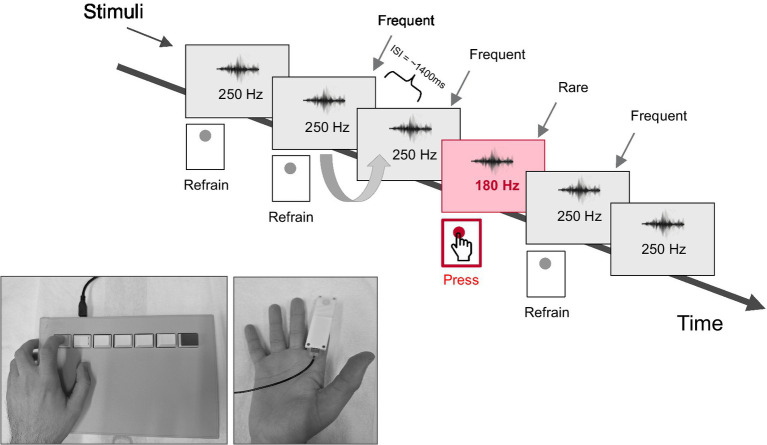
The vibrotactile oddball discrimination task and sequence of events. The stimuli lasted for 100 ms and they were presented to the right index finger in a randomized order with the constraint that no two rare stimuli occurred in a row. The participants were instructed to press the response pad with the left index finger once they detected a rare stimulus.

The vibrotactile stimuli consisted of two sinus-shaped vibrations; a 250 Hz (frequent) vibration and a 180 Hz (rare) vibration, each with a duration of 100 ms. To avoid abrupt stimulus onset and offset, each vibration was modulated with a 50 ms ramp (fade-in and fade-out), applied using a raised-cosine (Hanning window). These vibrations were generated using MATLAB 2023a (The MathWorks Inc, 2023, Natick, MA, United States) and using a sampling rate of 44.1 kHz. These frequencies were selected because they fall within the somatosensory frequency response range documented in the literature (Cardon and Sharma, 2018; Levänen and Hamdorf, 2001). The stimuli were delivered using a vibratory piezoelectric stimulator (Dancers Design) attached to the participants’ right index finger using a medical tape (as shown in Figure 1) and connected directly to a personal computer’s audio output.

The experiment consisted of an oddball tactile frequency change detection task in which participants were required to identify the rare stimuli within a series of frequent stimuli. Both the rare and the frequent stimuli were presented in a pseudo-randomized order with the constraint that no two rare stimuli occurred in a row. The presentation ratio of frequent to rare stimuli was 80:20. In total, 320 trials for the frequent condition (250 Hz) and 80 trials for the rare condition (180 Hz) were administered. All stimuli were delivered with a fixed inter-stimulus interval (ISI) of 1,400 ms.

The participants were seated comfortably in a quiet, well-lit, and sound proof room. To minimize ocular artifacts, they were instructed to close their eyes during the task. The participants were also instructed to respond as fast as possible to the rare stimuli by pressing the left response key with their left index finger. To ensure the stimuli were processed exclusively through the somatosensory pathway and were not perceived as auditory signals, participants wore earplugs throughout the task. All participants confirmed that while they could clearly perceive the vibrotactile stimuli on their right fingertip, they were unable to hear the auditory signal from the piezoelectric stimulator. Vibration intensity was individually adjusted for each participant to ensure it was clearly perceptible but did not cause discomfort. Prior to the main experiment, all participants completed a training session to ensure they could accurately discriminate between the frequent and rare stimuli. The total recording time was ~10 min (excluding breaks). Stimulus presentation was controlled using Presentation software V.23.1 (Neurobehavioral Systems, Inc., Berkeley, CA, United States).

### 2.3 EEG data acquisition and analysis

#### 2.3.1 Behavioral data: somatosensory oddball task

The percentage of hits (hit rate) and individual mean response times (RT) for correct trials were analyzed. Correct responses were defined as the occurrence of a button press in response to rare stimuli from 100 to 1,400 ms following stimulus onset. The behavioral analysis included four response types: hits, false alarms, misses, and correct rejections. Hits were defined as trials in which participants correctly responded to the rare stimulus, while misses were rare stimuli in which participants failed to respond. False alarms occurred when participants incorrectly responded to a frequent stimulus, and correct rejections were trials in which participants appropriately withheld responses to frequent stimuli. Additionally, accuracy (calculated as the percentage of correct responses, including both hits and correct rejections), mean reaction time (RT) for hits and its standard deviation were calculated. Since the relative frequencies of these response types are interdependent, hits and misses sum to one, as do false alarms and correct rejections. Additionally, we computed *d*-prime (*d*’) and log *β*, two key metrics within the framework of Signal Detection Theory (SDT) (Green and Swets, 1966). SDT provides a framework for assessing perceptual decision-making by estimating two essential parameters: *d*’, which quantifies the strength of the signal relative to background noise, and log β, which reflects the participant’s response strategy or bias in decision-making.

#### 2.3.2 Electrophysiological recording and data pre-processing

Continuous EEG was recorded using 64 channels with electrodes placed across the head according to the international 10–20 system (Klem et al., 1999), using a Neuroscan Quick-Cap with sintered Ag/AgCl electrodes (Neuroscan, Compumedics USA, Charlotte, NC, United States). A midline electrode placed between the Cz and CPz electrodes served as the online reference channel. Signals were amplified via a SynAmps RT system. Data were recorded with a sampling rate of 1,000 Hz and the electrode impedances were kept below 10 kΩ. To bypass delays associated with the stimulus presentation on the computer and accurately measure trigger timing at the onset of the stimulus, a Cedrus StimTracker was used to generate trigger pulses at stimulus onset. This ensures accurate timing of the stimuli. Following recording, EEG data was imported and analyzed with EEGLAB version 2024.2 (Delorme and Makeig, 2004) in the MATLAB environment (R2023a; Mathworks). The data were downsampled to 500 Hz to reduce data size and processing load. The data was then filtered offline using a FIR bandpass filter. The high pass cut-off frequency was 0.1 Hz with a maximum possible transition bandwidth of 0.2 Hz (two times cut-off frequency), and the low pass cut-off frequency was 40 Hz with a transition bandwidth of 2 Hz. For both cut-off frequencies, the Kaiser-window (beta = 5.653, maximal stopband attenuation = −60 dB, maximal passband deviation = 0.001) approach was used (Widmann et al., 2015). This approach maximizes the energy concentration in the main lobe, thus averaging out noise in the spectrum and reducing information loss at the edges of the window (Widmann et al., 2015).

Noisy channels were flagged, removed and replaced with interpolated data from remaining electrodes using a spline interpolation algorithm. We defined noisy channels as follows: either with a high impedance exceeding 30 kΩ or those having amplitudes greater than 100 μV. On average, 1.5 ± 0.5 channels per subject were interpolated in the SSD group and 1.27 ± 0.86 channels in the NH group. For removal of eye-artifacts, as well as muscle artifacts, we performed an independent component analysis (ICA) (Jung et al., 2000), as implemented in EEGLAB [RUNICA, (Delorme and Makeig, 2004)] over the entire continuous EEG data. We visually inspected component topographies, time courses and corresponding EEG segments. ICA components reflecting eye, muscle movements and alpha rhythm were discarded. The data was then segmented into epochs from −200 to 1,000 ms relative to the onset of vibration, and a baseline correction was applied (−200 to 0 ms). Individual epochs with amplitudes greater than 100 μV were excluded. The average number of epochs included for the frequent condition was 314.7 ± 7.7 for the SSD group and 312.2 ± 19.4 for the NH group. For the rare condition, the average number of epochs was 78.2 ± 3.1 for the SSD group and 77.7 ± 5.5 for the NH group. The data were then re-referenced to a common average reference before being exported for subsequent statistical and source analysis.

### 2.4 Statistical analysis

Statistical analysis was performed in R (Version 4.4.2, R Core Team, 2020, Vienna, Austria) and Curry 8 (Compumedics NeuroScan, Hamburg, Germany). We used the Wilcoxon Signed Rank test to compare age, air-conduction 4PTA, age at onset of deafness and duration of deafness between individuals with RSSD and LSSD. The chi-squared test was applied to compare sex between individuals with RSSD and LSSD. To explore potential associations between behavioral performance and participant characteristics, correlation analyses were conducted separately for the SSD and NH groups. This was done either by Spearman’s rank-order or Pearson’s correlation depending on the distribution of the data. In addition, supplementary correlation analyses (reported in Supplementary Table 3) were performed between the amplitudes of ERP components and the participant characteristics (age, duration of deafness, and audiometric thresholds). This additional comparison aimed to investigate whether individual variability in clinical and demographic factors was related to differences in neural responses, thereby offering insights into the extent to which cortical somatosensory processing may be influenced by auditory deprivation history. To address our different research questions, the amplitudes and peak times of CSEPs were analyzed separately on the sensor level (Left Parietal ROI on head surface: P50, N70, P100 and N140; parieto-central ROI on head surface: P3b component) and on the source level (ERP source analysis: activation in ipsi- and contralateral somatosensory cortices at P50 peak time). Additionally, a subgroup analysis was conducted to compare differences in peak amplitudes, peak times and source activation patterns between participants with LSSD and RSSD.

#### 2.4.1 ERP analysis

Only correct responses (hits for rare stimuli and correct rejections for frequent stimuli) were included in the ERP analysis. The rare stimulus epochs were used for analyzing the P3b component, while the frequent stimulus epochs were used for analyzing the P50, N70, P100, and N140 components. Both trial types were included in within-group comparisons to assess the modulation of somatosensory responses by stimulus frequency. This approach allowed us to examine condition-specific amplitude differences within each group. ERP averaging was performed separately for each subject and condition. For each subject, artifact-free epochs were averaged to generate individual ERP waveforms. These waveforms were then averaged across participants within each group to produce grand average waveforms.

The ROI for the initial CSEP analysis comprised of electrodes positioned over the left parietal scalp region. The selection of ROIs was guided by previous findings on optimal CSEP recording sites (Hämäläinen et al., 1990; Staines et al., 2014; Cardon and Sharma, 2018) and cortical regions identified as active during current density reconstruction (CDR) (Cardon and Sharma, 2018), specifically the parietal cortices, which correspond to somatosensory processing areas. The analysis for the right hemisphere is provided in the Supplementary material. The left parietal ROI (LPar) included the channels P1, P3, P5, P7, CP1, CP3, CP5, and the right parietal ROI (RPar) the channels P2, P4, P6, P8, CP2, CP4, and CP6. These ROI were used to analyze the P50, N70 P100 and N140 components. Regarding the P3b component, we used a parieto-central ROI which included the channels Pz, Cz, and CPz.

For CSEP quantification, individual peak amplitudes and peak times were measured by detecting the maximum amplitude and peak time of ERP peaks within commonly used latency bands for the P50, N70, P100, N140, and P3b components (Hämäläinen et al., 1990; Staines et al., 2014; Cardon and Sharma, 2018). These peak time ranges were selected based on both established literature (Johnson et al., 1980; Hämäläinen et al., 1990; Staines et al., 2014) and the occurrence of peaks in the grand average CSEP, ensuring that the time windows accurately captured component-specific neural responses. The selected ranges were: P50: 40–60 ms; N70: 60–95 ms; P100: 90–130 ms; N140: 130–175 ms; and P3b: 300–800 ms (Johnson et al., 1980; Hämäläinen et al., 1990; Staines et al., 2014).

Grand average CSEPs were computed for each ROI by first averaging the waveforms of all electrodes within the ROI for each condition. From these averaged waveforms, peak times and amplitudes were extracted. To obtain a group-level representation, each participant’s ROI-averaged waveform was further averaged to create the grand average waveform. An adaptive peak amplitude and peak time calculation was performed. The mean peak amplitude and peak time of each component were determined within the predefined time window on the grand average waveform. To refine individual peak detection, the group-level mean peak time was used to define an adjusted search window by adding or subtracting ±10 ms from this mean peak time. Within this adjusted time window, the maximum amplitude was identified for each individual. This extracted peak amplitude for each individual was subsequently used for statistical comparisons. For positive peaks (P50, P100, and P3b), the maximum amplitude within the adjusted time window was selected, whereas for negative peaks (N70 and N140), the minimum amplitude was extracted. In the case of P3b analysis, a broader adjustment range of ±30 ms was applied to ensure accurate peak detection. In the analysis of the P3b component, it is crucial to account for baseline neural activity to ensure that observed differences truly reflect cognitive processing related to target detection, rather than global fluctuations in neural excitability. To achieve this, frequent condition amplitudes were subtracted from rare condition amplitudes, yielding a difference waveform that isolates target-specific processing effects (Polich, 2007; Luck, 2012).

Peak amplitude and time values were then used in within- and between-group statistical comparisons in order to assess the differences between conditions and groups, respectively. Given that EEG data were not normally distributed, non-parametric Mann–Whitney *U* Tests were used to compare peak amplitudes and peak latencies. Post-hoc power analysis was conducted using G*Power (version 3.1.9.7). Multiple comparisons were corrected using the False Discovery Rate correction method introduced by Benjamini and Hochberg (1995).

#### 2.4.2 ERP topographical analyses in CSEP

To statistically assess differences in scalp topographies and underlying neural processes between groups and conditions, we employed a permutation-based, non-parametric Topographic Analysis of Variance (TANOVA) (Koenig and Melie-García, 2010). TANOVA is particularly advantageous as it identifies significant differences in scalp topographies independent of ERP amplitude, providing an unbiased statistical approach that is not influenced by the choice of reference electrode or predefined regions of interest. We first computed the global field power, which quantifies the spatial standard deviation of scalp potentials at each time point, reflecting the overall strength of the electric field. Global field power was used to assess the temporal stability and robustness of observed effects and to restrict statistical analyses to periods of high neural synchrony. TANOVA analyses were performed using Curry 8 software to evaluate within- and between-group comparisons for CSEP components (P50, N70, P100, N140, and P3b). To account for multiple comparisons, we applied a corrected significance threshold based on the alpha level (0.05), sampling rate of the data, and low-pass filter frequency, resulting in an adjusted alpha level of 0.01 and randomization value of 6,117 to minimize false positives. Additionally, only effects lasting 20 ms or longer were considered statistically significant, ensuring the robust detection of meaningful differences in global field power analyses (Guthrie and Buchwald, 1991; Murray et al., 2008).

#### 2.4.3 Source level analysis – ERP source reconstruction

For source analysis, both frequent and rare stimulus epochs were combined to increase the number of epochs, thereby enhancing the signal-to-noise ratio (SNR) and improving the reliability of the source estimation. The P50 component, which reflects the direct cortical response of S1, was localized in both groups using the sLORETA algorithm, as implemented in Curry 8 software (Wagner et al., 2014). We investigated only this component because we were interested in changes in the primary somatosensory response, and also because the most prominent peak displayed on the Mean Global Field Power map, occurred between the time ranges 30–70 ms (indicating the range where most dipoles are likely to occur), and corresponding to that of the P50 CSEP. sLORETA employs specific mathematical algorithms to inversely calculate the intensity and three-dimensional spatial distribution of neuronal electric activity sources from the EEG data recorded on the scalp (Pascual-Marqui, 2002; Wagner et al., 2004). To test for significant differences in the source activation between groups, CDR statistical non-parametric mapping (SnPM) was used (Wagner et al., 2014).

The analysis followed a structured sequence of steps. First, ERP data were imported (EDF format) into Curry 8, and group-averaged. Following, epochs that presented with data exceeding ±100 mV in amplitude were also eliminated. Next, SNR detection was conducted by selecting the noise window as the pre-stimulus interval (−200 ms to 0) where no stimulus-driven brain activity is expected. A SNR greater than 10 was considered as valid. We obtained a SNR of 11.2 for NH controls and 13.1 for individuals with SSD. Principal Component Analysis was then applied, selecting the time window 30–70 ms for component analysis based on the average global field power, ensuring an SNR above 1 for valid data. The standard boundary element method head model was employed, derived from an averaged MRI dataset from the Montreal Neurological Institute (MNI) database (Fuchs et al., 2002). The modeling was constrained to brain structures, including only gray and white matter, while excluding the skin, skull, dura, and ventricles. To localize neural activity, a moving dipole approach was used, with dipole location determined at the peak of the averaged waveform. Dipole modeling takes the voltage value from all the electrodes at that given instant in time. It searches for the equivalent dipole within the head model that could be possible generators of the CSEPs, respectively (Cuffin, 1985). The resulting dipole analysis was then projected onto the head model. Dipole modeling utilized voltage values from all electrodes at a specific time point to identify the most likely dipole sources within the head model that could account for the observed neural activity (Henderson et al., 1975; Fuchs et al., 2002; Ebersole, 2009). The results of this method were represented as color gradients, illustrating the F-distribution of the data, and subsequently overlaid onto the MNI average brain template, ensuring standardized anatomical localization (Evans et al., 1993).

## 3 Results

### 3.1 Behavioral measures

Table 2 summarizes the results for all behavioral measures compared between both groups. Three participants with SSD and one NH participant were removed from the behavioral data analysis because of low hit rates near chance level, unusually slow RTs (greater than three times the standard deviation) and non-compliance with task instructions (e.g., participant never responded). Trials for which no response was given (NH: 18.3%, SSD: 17.9%) were excluded from the analysis. Additionally, trials with a RT higher than three standard deviations above the individual mean were removed (NH: 1.10%, SSD: 1.11%). As the data were not normally distributed (Shapiro–Wilk Test, *p* < 0.05), non-parametric statistics (Mann–Whitney *U*-test) were applied for the analyses. The effect sizes were computed using the following formula *r* = *Z*/√*N*, where *Z* is the *Z* score from the test and *N* is the total number of observations (Cohen, 2013). Across all behavioral metrics, the analysis revealed no significant differences between the NH and SSD groups. The effect sizes were small (*r* ≤ 0.1). Similarly, the comparison between the LSSD and RSSD groups showed no significant difference (see Supplementary Table 2).

**Table 2:** Behavioral performance measures for the normal hearing (NH) and single-sided deaf (SSD) groups.

	NH (*n* = 19)	SSD (*n* = 17)	*p*-value
	Median (min – max)	Median (min – max)	
Hit rates	71 (54–78)	70 (51–78)	0.924
False alarms	2 (0–5)	4 (1–8)	0.211
Misses	9 (2–26)	10 (2–29)	0.924
Correct rejections	318 (315–320)	316 (312–319)	0.211
Accuracy (%)	89 (68–98)	88 (64–98)	0.924
*d*’	3.36(2.59–4.24)	3.68 (2.05–4.27)	0.908
log *β*	1.22 (0.74–2.26)	1.53 (0.90–1.81)	0.961
Hit RT (ms)	493.91 (430.89–618.73)	495.32 (449.92–550.53)	0.392

*Significance level of 0.05* and of 0.01**. RT is for reaction time. SD is for standard deviation. Hit rates, false alarms, misses, correct rejections are reported as counts. *d*’ and log β are dimensionless statistical measures.

### 3.2 Waveform analysis

#### 3.2.1 Within-group ERP differences between conditions

**Figure 2:**
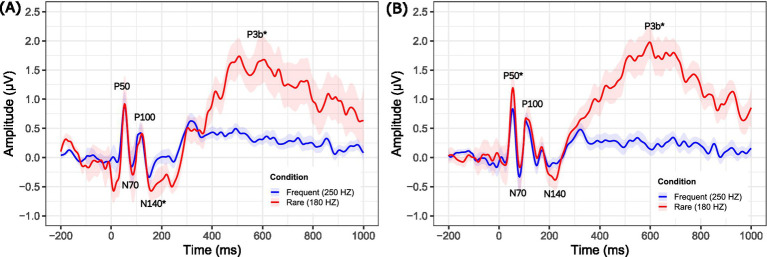
Grand average cortical somatosensory evoked potentials (CSEPs) responses to frequent (250 Hz) and rare (180 Hz) vibrotactile stimuli in the NH (**A**, *n* = 20) and SSD (**B**, *n* = 20) groups at the left parietal (Lpar) region of interest. The solid red line represents the response to frequent stimuli while the solid blue line represents the response to rare stimuli. Shaded areas around the waveforms indicate standard error of the mean. Key CSEPs components (P50, N70, P100, N140, and P3b) are labeled at their peak times. Significantly different CSEP peak amplitudes are denoted by asterisks—single asterisks indicate significance at the *p* = 0.05 level. The stimulus onset occurred at 0 ms and the stimulus lasted for 100 ms. The epoch window shown spans from −200 ms (pre-stimulus) to 1,000 ms (post-stimulus).

Figures 2A,B illustrate the differences in amplitudes of CSEPs across stimulus conditions in the NH (left panel) and SSD (right panel) groups, for the Lpar ROI. A paired-Wilcoxon signed-rank test was conducted to compare the maximum amplitudes of CSEPs across stimulus conditions within each group (NH and SSD). In the NH group, a significant difference was found between the frequent (250 Hz) and rare (180 Hz) stimuli for the N140 component (*W* = 178, *p* = 0.0194) and P3b (*W* = 3, *p* = 0.0010). Effect sizes (Cohen’s *r*) were *r* > 1 for N140 and *r* = 0.67 for P3b, both indicating large effect sizes. In the SSD group, significant differences were observed between the frequent (250 Hz) and rare (180 Hz) stimuli for P50 (*W* = 24, *p* = 0.008) and P3b (*W* = 3, *p* = 0.001). Effect sizes were *r* > 1 for P50 and *r* = 0.67 for P3b, both indicating large effect sizes. Significant results for the Rpar ROI can be found in the Supplementary Figure 1.

#### 3.2.2 Differences in ERPs’ peak amplitudes and peak times between groups

##### 3.2.2.1 Early stages of somatosensory processing (frequent stimulus processing)

**Figure 3:**
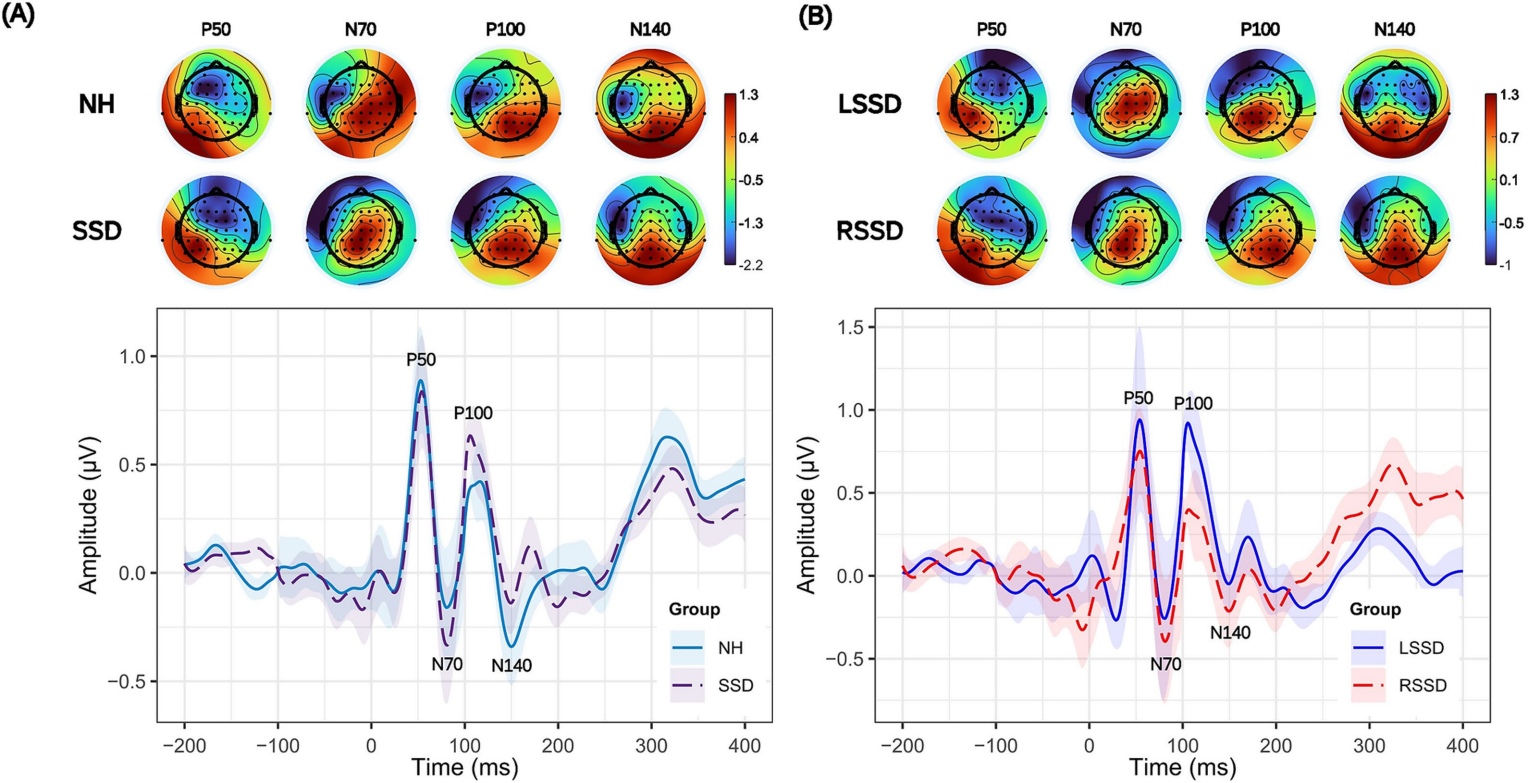
Grand average cortical somatosensory evoked potentials (CSEPs) and topographical scalp distributions to the frequent tactile stimulus. The left panel **(A)** compares CSEP responses between NH (blue solid line) and SSD (dashed violet line) at the left parietal region of interest (ROI). The right panel **(B)** compares the CSEP responses between individuals with left single-sided deafness (LSSD, *n* = 9, blue solid line) and right single-sided deafness (RSSD, *n* = 11, red dashed line) for the left parietal ROI. The shaded areas represent the standard error of the mean. The key somatosensory evoked potential (CSEP) components—P50, N70, P100, and N140—are labeled.

Figures 3A,B presents differences in the P50, N70, P100, and N140 components of the CSEP at the Lpar ROI. Visual inspection of the grand average waveform morphology indicates that the NH group exhibits smaller peak amplitudes for N70 and P100 compared to individuals with SSD, even though it did not reach statistical significance. A subgroup analysis further examined differences between participants with LSSD and RSSD. Figure 3B illustrates an increase in P50 and P100 peak amplitudes in the LSSD subgroup, which also did not reach statistical significance. The effect sizes were low (r ≤ 0.2) and statistical power for detecting differences were below 50%.

##### 3.2.2.2 Later stages of somatosensory processing (rare stimulus processing)

**Figure 4:**
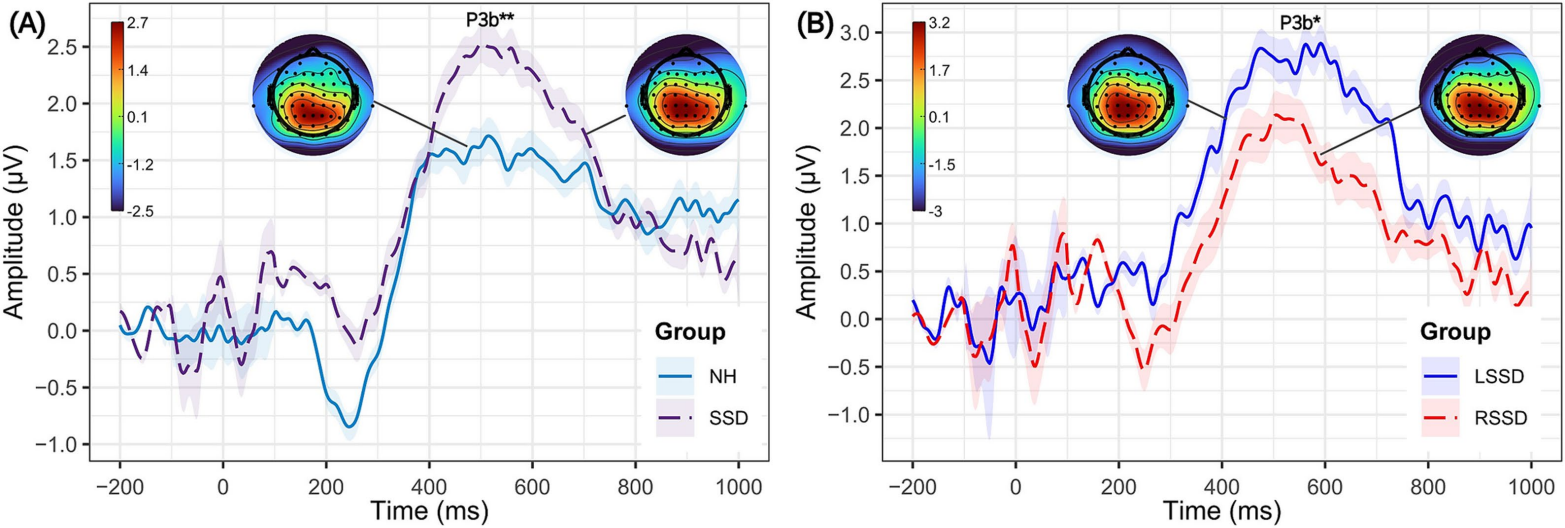
Grand average cortical somatosensory evoked potentials (CSEPs) and topographical scalp distributions to rare tactile stimulus. The left panel **(A)** compares P3b of CSEP responses between individuals with NH (solid blue line) and SSD (dashed violet line) at the centro-parietal region of interest (ROI). The right panel **(B)** compares the P3b CSEP responses between individuals with left single-sided deafness (LSSD, *n* = 9, solid blue line) and right single-sided deafness (RSSD, *n* = 11, dashed red line), for the centro-parietal ROI. The shaded areas represent the standard error of the mean. Significantly different CSEP peak times are denoted by asterisks—single asterisks indicate significance at the *p* = 0.05 level, while double asterisks highlight differences at the *p* = 0.001 level.

The ERPs for the somatosensory oddball task, separately for NH and SSD groups (left panel), and LSSD and RSSD groups (right panel), are depicted in Figure 4. Three participants with SSD and one NH participant who demonstrated difficulties performing the oddball task were removed from the analysis. The grand average waveform at the parieto-central ROI showed a positive peak between 300 and 800 ms in response to vibrotactile stimuli (referred to as P3b) (Ritter and Vaughan, 1969; Polich, 2007). Effect size for P3b peak time comparison was large (*r* > 0.8), resulting in greater statistical power (>85%). The mean peak amplitudes for the P3b CSEP component in the NH, SSD, LSSD, and RSSD groups are shown in Figures 4A,B respectively, while the mean peak times are reported in Table 3. A graphical representation of the P3b difference waveform can be found in the Supplementary Figures 3, 4.

**Table 3:** Mean cortical somatosensory evoked potential (CSEP) peak times and standard deviations for the normal hearing (NH), single-sided deafness (SSD), left single-sided deafness (LSSD) and right SSD (RSSD) groups.

CESP component	Group	Mean peak time (ms)	Std. deviation	95% confidence interval (lower – upper bound)	Statistic (Mann–Whitney U)	*p*-value
P3b	NH	514.0	21.61	503.88–524.11	35.5	<0.001**
	SSD	556.5	21.83	546.28–566.71		
	LSSD	573.3	36.40	545.35–601.31	71	0.032*
	RSSD	536.0	24.58	518.41–553.58		

*Significance level of 0.05* and of 0.01**.

Visual inspection of waveform morphology shows the P3b component to be larger in the SSD group compared to the NH group. However, statistical analysis revealed no significant difference in maximum amplitude between SSD (*M* = 2.88 μV, SD = 1.82 μV) and NH (*M* = 2.20 μV, SD = 1.81 μV) groups (*U* = 146, *p* = 0.147). In contrast, statistical analysis confirmed a significant delay in P3b peak time in individuals with SSD (*M* = 556.5 ms, SD = 21.8 ms) compared to NH controls (*M* = 514.0 ms, SD = 21.6 ms) (*U* = 35.5, *p* < 0.001), suggesting prolonged processing time in individuals with SSD. The effect size was calculated using the rank biserial correlation and was found to be small (*r* < 1). A subgroup analysis further examined differences between participants with LSSD and RSSD. While mean P3b amplitude was higher in LSSD (*M* = 3.33 μV, SD = 1.60 μV) compared to RSSD (*M* = 2.75 μV, SD = 1.99 μV), this difference was not statistically significant (*p* = 0.594). However, a significant difference in P3b peak time was observed, with LSSD showing a longer peak time (*M* = 573.3 ms, SD = 36.4 ms) compared to RSSD (*M* = 536.0 ms, SD = 24.6 ms) (*U* = 71, *p* = 0.032). The effect size *r* = 0.43, indicating moderate effect size.

**Figure 5:**
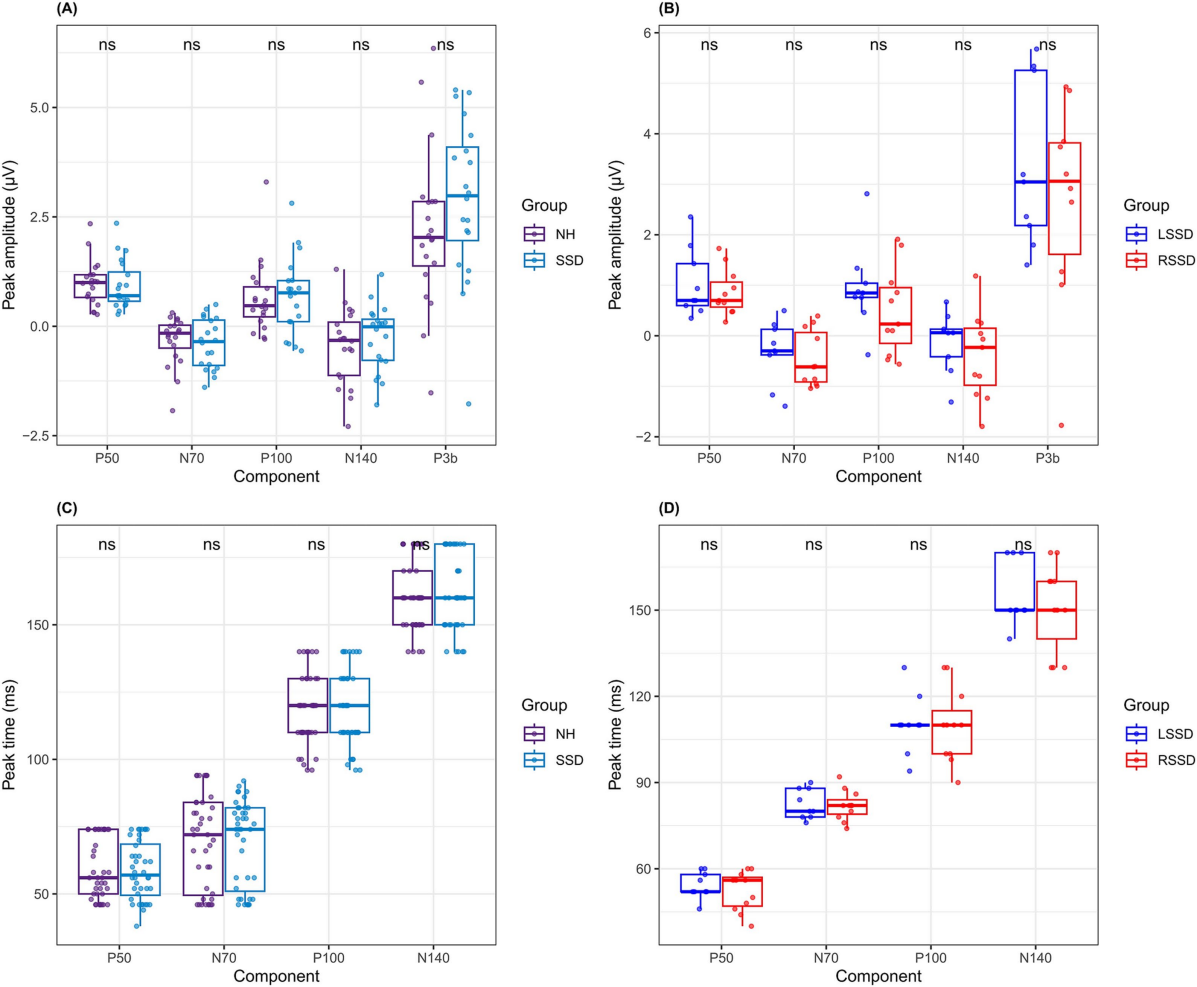
Median cortical somatosensory evoked potential (CSEP) peak amplitudes and standard deviations for **(A)**: the normal hearing (NH), **(B)**: single-sided deafness (SSD), **(C)**: left single-sided deafness (LSSD) and **(D)**: right SSD (RSSD) groups.

Figures 5A–D summarizes the mean peak amplitudes and peak times for each CSEP component in each group and subgroup. Additional information concerning the peak amplitudes and peak times for the right parietal ROI is available in the Supplementary Figure 2.

#### 3.2.3 Topographical analysis of ERP components

A TANOVA was conducted to compare topographical differences of the CSEPs between groups (NH vs. SSD) and conditions (frequent vs. rare). Within-group TANOVA results revealed significant topographic differences between the frequent and rare conditions in both NH and SSD groups during the P3b time window, with the following significant intervals: 180–518 ms, 556–730 ms, 336–376 ms, 584–652 ms, and 848–996 ms (*p* < 0.001). The rare stimuli elicited distinct neural responses within these peak times, and displayed the largest positivity over central and parietal electrodes, with activity predominantly localized to the left hemisphere. No significant differences between groups were found for the peak times of the early exogenous ERP components P50, N70, P100, N140 and endogenous P3b component.

### 3.3 Source analysis

**Figure 6:**
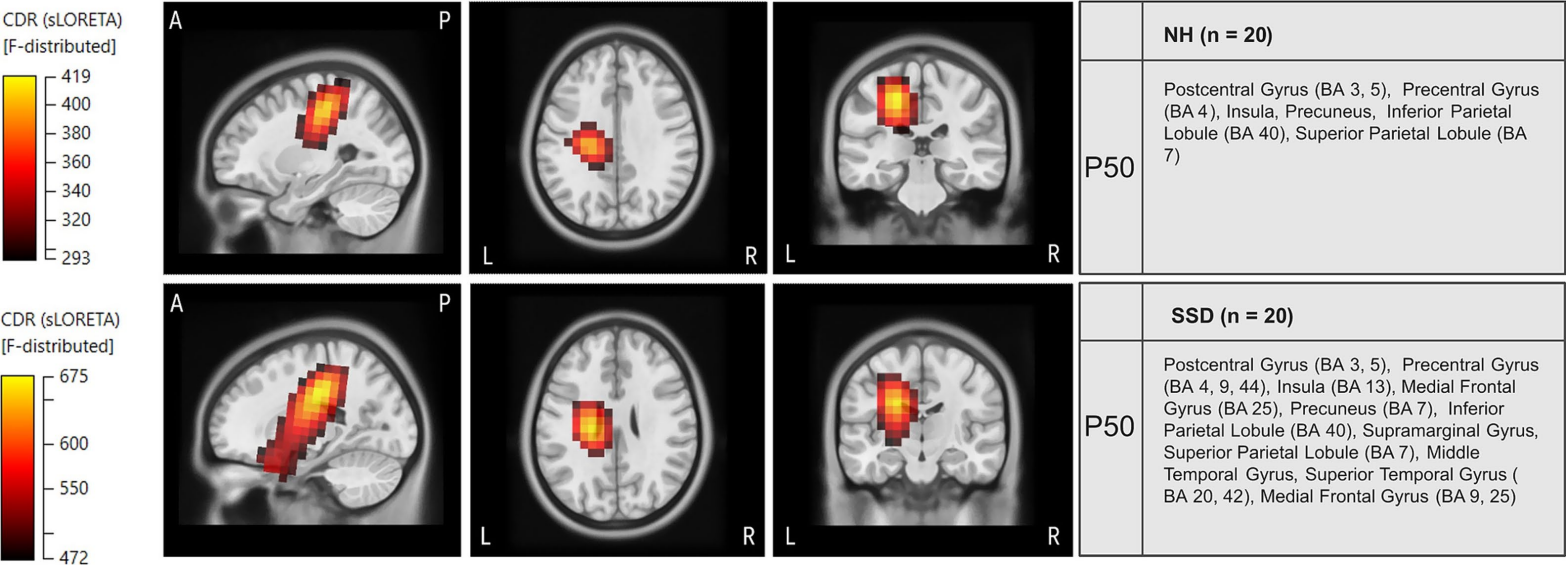
Current density source reconstructions (CDR) of the P50 cortical somatosensory evoked potential (CSEP) component in the normal hearing (NH) and single-sided deafness (SSD) groups. The top row represents CDRs for the NH group, while the bottom row represents CDRs for the SSD group. Cortical source reconstructions were obtained using standardized low-resolution brain electromagnetic tomography (sLORETA) and projected onto a Montreal Neurological Institute (MNI) template in sagittal, axial and coronal views. The color scale represents the statistical likelihood of activation based on an F-statistic, with darker red indicating lower probability and brighter yellow indicating the highest probability. The table on the right lists the brain regions exhibiting the highest cortical source activity for the P50 CSEP component. Activations are shown for the left hemisphere.

**Figure 7:**
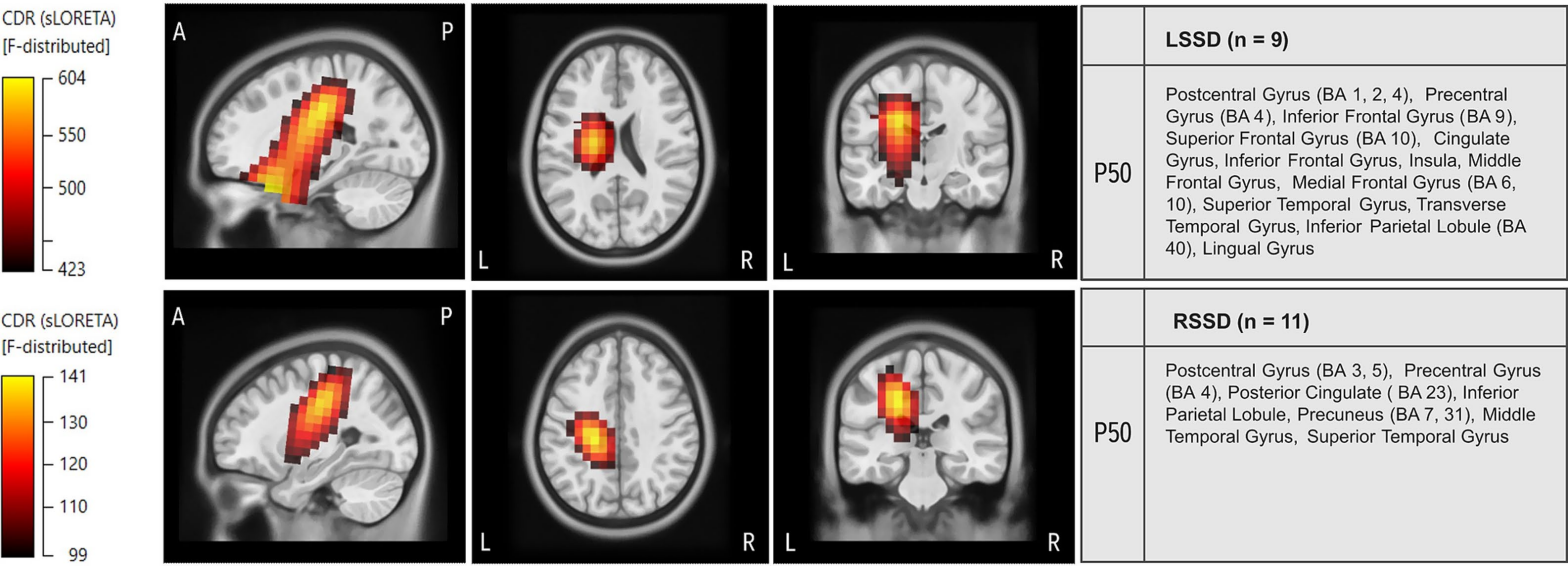
Current density source reconstructions (CDR) of the P50 cortical somatosensory evoked potential (CSEP) component in left single-sided deafness (LSSD) and right single-sided deafness (RSSD) groups. The top row represents CDRs for the LSSD group, while the bottom row represents CDRs for the RSSD group. Cortical source reconstructions were obtained using standardized low-resolution brain electromagnetic tomography (sLORETA) and projected onto a Montreal Neurological Institute (MNI) template in sagittal, axial and coronal views. The color scale represents the statistical likelihood of activation based on an F-statistic, with darker red indicating lower probability and brighter yellow indicating the highest probability. The table on the right lists the brain regions exhibiting the highest cortical source activity for the P50 CSEP component. Activations are shown for the left hemisphere.

Current density reconstruction (CDR) was performed for the P50 CSEP. The resulting current density distribution across the sagittal, axial and coronal planes is depicted in Figures 6, 7.

In the NH group, CDR analysis revealed a clear activation in the contralateral (left) somatosensory cortex, specifically in the postcentral gyrus (Brodmann Areas, BA 3 and BA 5), as well as the precentral gyrus (BA 4), the inferior parietal lobule (BA 40), and the superior parietal lobule (BA 7). These contralateral activations were expected based on the decussation of ascending somatosensory pathways. The specific area in the S1 that represents the fingers is located in BA 3b, BA 1 and part of BA 2 (considered as primary areas for tactile processing receiving direct input from the thalamus: Ventral Posterolateral Nucleus) (Kaas, 1993; Penfield and Boldrey, 1937).

Similarly, the SSD group exhibited activation in the motor and somatosensory cortices. However, in contrast to the NH group, the SSD group displayed additional activation in regions not typically associated with somatosensory processing (see Figure 6). These regions included the Middle Temporal Gyrus (MTG), the Superior Temporal Gyrus (STG, BA 20, and BA 42), and the Medial Frontal Gyrus (BA 25, BA 9).

Figure 6 shows the CDR of P50 in the LSSD and RSSD groups. The results reveal distinct activation profiles between the two subgroups. The LSSD group exhibited greater recruitment of auditory and multimodal processing areas, including the STG, the Transverse Temporal Gyrus and the Insula. Increased activation was found in language-related regions (Labache et al., 2019) like the Inferior Frontal Gyrus (BA 9, 10) and the Middle Frontal Gyrus. Additionally, higher current density was observed in the Lingual Gyrus, the Medial Frontal Gyrus (BA 6, BA 10), and the Cingulate Gyrus, suggesting increased engagement of higher-order cognitive regions. The Postcentral Gyrus (BA 1, BA 2, BA 4), the Precentral Gyrus (BA 4), and the Inferior Parietal Lobule (BA 40) were also activated.

In contrast, the RSSD group exhibited more localized activation in primary somatosensory and auditory areas, particularly the Postcentral Gyrus (BA 3, BA 5), the Precentral Gyrus (BA 4), and the Posterior Cingulate (BA 23). Additionally, higher activity in the Precuneus (BA 7, BA 31), the Middle Temporal Gyrus and the STG suggests that RSSD individuals may rely more on somatosensory processing pathways, with less pronounced auditory cortical recruitment compared to LSSD.

**Figure 8:**
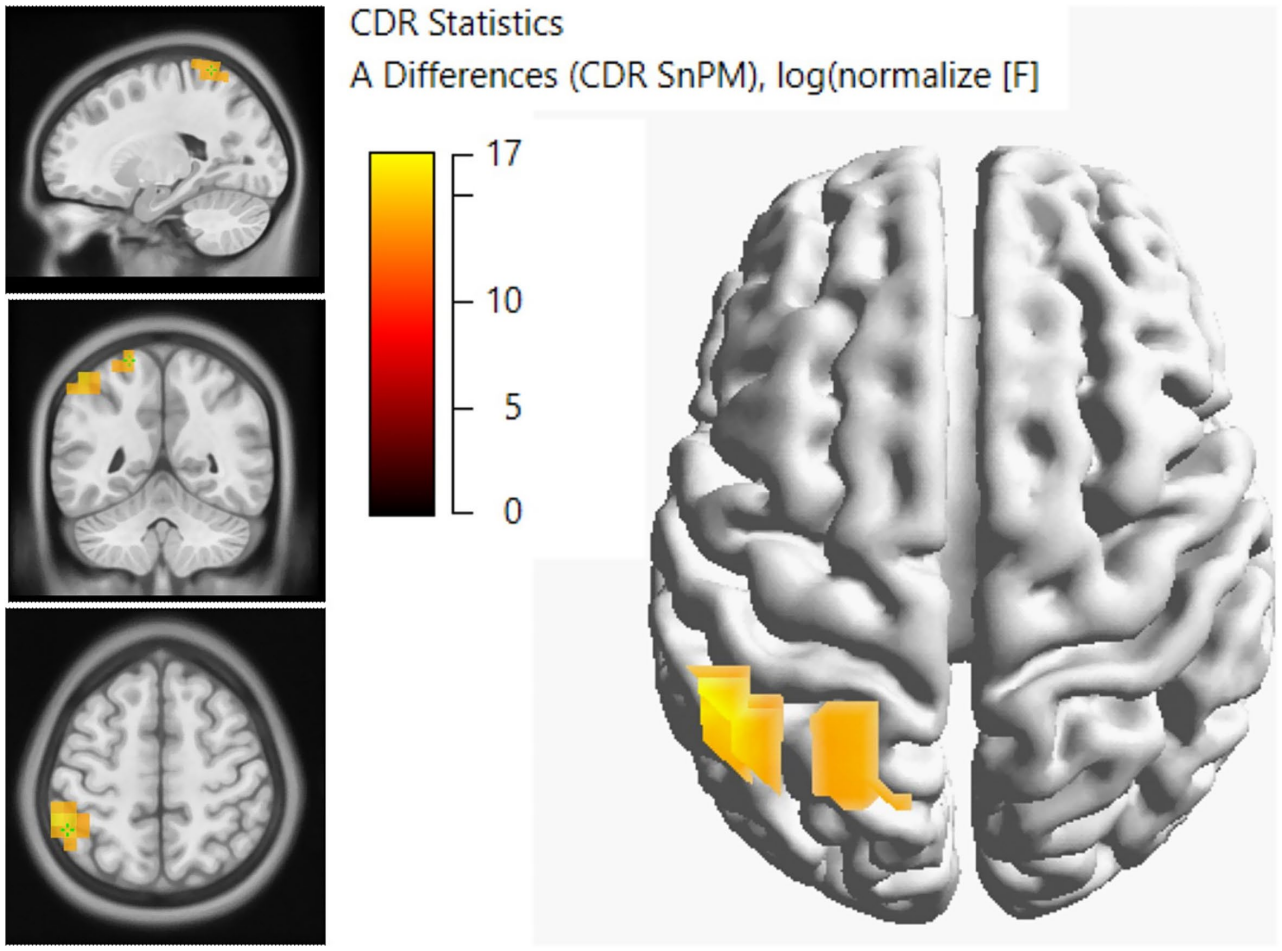
Displays results of the current density reconstruction statistical nonparametric mapping (CDR SnPM) testing between the normal hearing (NH) controls and individuals with single-sided deafness (SSD). The input data for the CDR SnPM were the standardized Low Resolution Brain Electromagnetic Tomography (sLORETA) source images for the rare conditions in each group. Source analysis revealed that individuals with SSD recruited more parietal and auditory cortices during somatosensation.

To validate this source difference between groups, additional sLORETA analysis for the P50 interval (30–70 ms) was computed for the rare condition. Our, results showed a significant contiguous segment (*p* < 0.05) from 42.0 to 54.0 ms (7 samples); predominantly on the parietal cortex (left Postcentral Gyrus, the Left Inferior Parietal Lobule; BA 40, the left Superior Parietal Lobule; BA 7) and auditory cortex (the Left Middle Temporal Gyrus) in the SSD group (see Figure 8). No significant differences was found between LSSD and RSSD.

## 4 Discussion

In this study, we investigated whether adults with acquired SSD exhibit evidence of somatosensory cross-modal reorganization compared to NH adults and whether this reorganization differs between individuals with LSSD and RSSD. Despite the absence of significant differences in behavioral performance between groups (NH vs. SSD), our findings indicate that individuals with acquired SSD exhibit alterations in attentional mechanisms associated with somatosensory perception. While P3b amplitudes were increased in individuals with acquired SSD, this difference did not reach statistical significance. However, individuals with acquired SSD demonstrated significantly delayed P3b peak times compared to age- and sex-matched NH controls, suggesting prolonged cognitive processing in response to somatosensory stimuli. Source reconstruction analyses further revealed greater activation in auditory cortices (including the STG, the Middle Temporal Gyrus, and the Medial Frontal Gyrus) in individuals with acquired SSD during vibrotactile stimulation. Subgroup analysis showed that individuals with LSSD – when compared to RSSD – recruited more auditory-associated cortical areas, particularly the STG, the Transverse Temporal Gyrus, and the Insula. This further supports the notion of compensatory neuroplasticity in auditory pathways following severe to profound unilateral hearing loss.

### 4.1 Behavioral measures

For all behavioral performance measures, no significant differences were observed between the NH and SSD groups. These findings suggest that acquired SSD does not impair or improve performance in the somatosensory discrimination task, indicating that basic somatosensory perception, decision-making accuracy, and response speed remain comparable to NH individuals. Previous studies have reported mixed results regarding the behavioral impact of auditory deprivation on non-auditory tasks. Some research suggests that varying degrees of hearing impairment lead to compensatory enhancements in visual and somatosensory processing, potentially caused by cross-modal plasticity (Bavelier and Neville, 2002; Hauthal et al., 2015; Hennesy et al., 2022). In contrast, other studies in cochlear implant users indicate that while neural reorganization occurs, it does not necessarily translate to behavioral changes (Doucet et al., 2006; Heimler et al., 2014). Our findings are comparable to those of Levänen and Hamdorf (2001), who investigated tactile frequency discrimination in individuals with congenital bilateral deafness. Their study assessed whether participants could determine if a frequency-modulated test stimulus (ranging from 160 to 250 Hz) was increasing or decreasing in frequency relative to a fixed 200-Hz reference stimulus. Like our study, their results indicated no significant difference in discrimination ability between individuals with congenital bilateral deafness and NH controls. Unlike our results, they however observed that individuals with congenital bilateral deafness exhibited greater sensitivity compared to individuals with NH when asked to detect changes between a frequent standard 250-Hz stimulus and a rare 180-Hz stimulus. Age-related differences in vibrotactile perception have been documented (Frisina and Gescheider, 1977; Chen et al., 2019). Research comparing children and adults revealed that at frequencies above 200 Hz, discrimination thresholds were virtually identical for both groups. However, below this frequency, children were more sensitive than adults (Frisina and Gescheider, 1977). In the present study, the response accuracy and age were not significantly correlated, neither in individuals with SSD nor in NH controls. Taking into consideration the electrophysiological findings and ERP source analysis results of our study, we can conclude that even though the somatosensory behavioral performance remains preserved in individuals with acquired SSD, the maintenance requires adaptive neural plasticity and the recruitment of alternative neural pathways.

### 4.2 Electrophysiological measures

#### 4.2.1 Differences at earlier exogenous CSEP peak times (P50, N70, P100, N140)

Firstly, we observed a significant difference in P50 in individuals with acquired SSD between frequent (250 Hz) and rare (180 Hz) stimulus conditions. The rare stimuli elicited a larger P50 component than the frequent stimuli, suggesting altered early-stage exogenous cortical encoding of vibrotactile frequency information. This condition difference in the P50 response was not evident in NH controls in the present study. A future study with reverse stimulus roles will help us to disentangle whether the observed differences are attributable solely to stimulus presentation probability or whether they may partially reflect physical differences between the stimuli. A previous study on NH individuals observed that low-frequency somatosensory stimuli are often detected more effectively than stimuli with higher frequencies (Choi et al., 2017). Indeed, a study examining discrimination thresholds for vibrotactile stimulation at various high frequencies found significant differences, particularly between 150 Hz and both 200 and 225 Hz. The lower perceptual thresholds at 200 Hz and 225 Hz suggest heightened sensitivity to these frequencies (Choi et al., 2017). This difference is largely caused by a differential activation of the mechanoreceptor and the central processing mechanism. Nangini et al. (2006), while working with NH individuals demonstrated that transient onset responses in the lower frequency band (<20 Hz) resulted in a clearly expressed P50 component, whereas higher frequency bands (18–30 Hz) revealed different response patterns, such as gamma-band responses and steady-state responses. Therefore, the observed enhanced P50 response to low-frequency stimulation in individuals with acquired SSD may be caused by the differential processing characteristics of the somatosensory system. Lower frequency stimuli may engage primary somatosensory cortex neurons more effectively, leading to a more robust P50 component. In contrast, higher frequency stimuli tend to produce steady-state responses, reflecting continuous processing rather than the transient response associated with the P50 component. Another study from Park et al. (2021) who examined neural coding of vibration intensity in NH individuals found that low frequency vibrotactile stimuli are more effective in eliciting robust P50 CSEP compared to higher frequency stimuli. In an MEG study using similar frequencies, Levänen et al. (1998) found out that adults with acquired, bilateral deafness could use the auditory cortices to discriminate between the applied 180 Hz and 250 Hz frequencies. Levänen et al. (1998) also showed that only in individuals with congenital, bilateral deafness, but not in the NH controls, the vibration-induced S1 activation was followed by a strong bilateral activation of the supratemporal (ST) auditory cortices.

Secondly, individuals with acquired SSD exhibited no significant differences in amplitudes and peak times for early exogenous CSEP components (P50, N70, P100, N140) compared to NH controls (see Figure 5). Furthermore, no significant differences were found between LSSD and RSSD, suggesting that early-stage somatosensory processing remains functionally preserved in individuals with acquired SSD, regardless of the side of auditory deprivation. These findings align with previous research indicating that the absence of binaural auditory input does not necessarily alter the early exogenous cortical processing of tactile stimuli (Levänen et al., 1998; Karns et al., 2012). Our results are also in accordance with those of Cardon and Sharma (2018) who found no significant differences in amplitudes and peak times between these components at the left parietal region of interest in patients with age-related early stage bilateral hearing loss.

#### 4.2.2 Electrophysiological differences at later endogenous CSEP peak times (P3b)

Additionally, our findings indicate that SSD induces alterations in higher-order cognitive functions and attention mechanisms associated with somatosensory perception, primarily reflected by an increase in P3b amplitudes during vibrotactile stimulation (Polich, 2007; Verleger, 2020). Although not statistically significant, this will be compatible with a possible increase in recruitment of neural resources in response to vibrotactile stimuli, potentially reflecting compensatory mechanisms following auditory deprivation. Furthermore, the significant delay in peak P3b times in the individuals with acquired SSD compared to NH controls suggests slower stimulus processing and decision-making speed. Subgroup analyses further revealed distinct patterns of neural adaptation in individuals with LSSD and RSSD. While P3b amplitudes were slightly higher in LSSD, this difference was not statistically significant. However, P3b peak times were significantly longer in LSSD compared to RSSD, suggesting prolonged stimulus processing in individuals with acquired SSD of the left ear.

The differences in neural processing observed in the later time windows between individuals with acquired SSD and NH controls in the present study suggest that hearing loss influences cognitive resource allocation, ultimately affecting post-perceptual stimulus processing in later stages of neural processing (Finke et al., 2015). The P3b component, occurring between 300 to 800 ms is often linked to cognitive processing and consciousness of perception (Ritter and Vaughan, 1969). Both the NH and SSD groups showed significant differences in the P3b components between frequent and rare conditions. This is consistent with the suggestion that both groups tends to recruit more cognitive resources to address processing involving rare stimuli compared to frequent stimuli. Our result that SSD shows larger P3b amplitudes than NH is in line with that of Hauthal et al. (2015) who found significantly larger tactile P3b amplitudes in adults with bilateral deafness compared to adults with NH. Moreover, González-Garrido et al. (2017) who used a much higher frequency of 700 Hz (rare stimuli) reported a significant P3b amplitude increase and behavioral improvement in individuals both with bilateral prelingual profound deafness and normal hearing, after short training periods. These findings indicate that vibrotactile discrimination training can lead to behavioral improvements and neural adaptations in individuals with profound bilateral hearing loss, enhancing their ability to process vibrotactile stimuli.

In examining potential factors known to influence cortical organization, we found no significant effect of age at examination, age at onset of deafness and duration of deafness on the P3b component. It is important to note, however, that age at onset of deafness and duration of deafness are inherently subjective and often challenging to determine accurately in individuals with SSD. In this study, the age at onset of deafness was defined as the time point at which the participant discontinued use of a hearing aid in the poorer-hearing ear because of a lack of perceived or audiometric benefit. Nevertheless, the variables have been proven to associate with neuronal activation (Lee et al., 2001, 2007; Han et al., 2019; Speck et al., 2020) and are widely used to predict outcome after treatment with CI (Van Dijk et al., 1999; Bodmer et al., 2007; Blamey et al., 2013; Kitterick et al., 2014; Savvas et al., 2020; Speck et al., 2022).

#### 4.2.3 Somatosensory cross-modal recruitment

Source analysis of the P50 component in individuals with acquired SSD revealed enhanced activation in cortical areas traditionally associated with auditory processing, including the STG, the Transverse Temporal Gyrus, and the Middle Temporal Gyrus. This neural reorganization could be an adaptive response to auditory deprivation, facilitating the integration of non-auditory sensory inputs to compensate for the loss of binaural auditory cues. Interestingly, individuals with LSSD exhibited greater recruitment of auditory-related cortical regions and language related areas, including the STG, Inferior Frontal Gyrus, Insula, and Middle Frontal Gyrus, while individuals with RSSD showed increased activation in somatosensory and multimodal integration areas, such as the Postcentral Gyrus, the Precuneus, and the Inferior Parietal Lobule.

Although not statistically significant, these findings suggest that LSSD leads to greater reliance on auditory-related regions, whereas RSSD engages more somatosensory-driven processing pathways. Indeed, differences between LSSD and RSSD regarding functional asymmetry have also been observed in auditory stimulus conditions (Weglage et al., 2022). Further, this lateralized effect may be attributed to differences in hemispheric specialization for sensory processing, with the left hemisphere playing a dominant role in speech perception (Zatorre and Belin, 2001) and the right hemisphere contributing more to spectral and spatial processing (Tervaniemi and Hugdahl, 2003).

The traditional view of the REA posits that the right ear, because of its direct neural pathways to the left, language-dominant hemisphere, provides faster auditory processing of language (Prete et al., 2018). This model predicts that loss of the “dominant” ear, particularly in those with REA leads to significant functional impairments. However, our findings in individuals with RSSD appear to contradict this prediction. Rather than demonstrating a significant loss in cognitive performance caused by the deprivation of the right ear’s input, we found that individuals with RSSD exhibited less widespread cross-modal activation compared to individuals with LSSD. This might be also caused by our choice to only simulate the right index finger (of the dominant hand) and therefore limit the resulting activation of the left hemisphere.

In individuals with LSSD, deficits in right hemisphere, specifically in the processing of spectral and spatial auditory cues appear to impede the intramodal computation of auditory and vibrotactile stimuli. As a result, these individuals engage in greater cross-modal activation, recruiting additional language-associated regions to compensate for deficits in their spectral processing. This need for cross-modal engagement is particularly notable in the context of language functions, where expanded neural networks are activated to process non-auditory input. It would be valuable to investigate how stimulating the non-dominant left hand, or both hands simultaneously, might influence our results.

Our source analysis findings align with evidence from Karns et al. (2012), who conducted an fMRI study demonstrating that in individuals with bilateral congenital deafness, Heschl’s gyrus exhibited greater activation in response to somatosensory and auditory-somatosensory (bimodal) stimuli compared to individuals with NH. This suggests that, in the absence of auditory input, traditionally auditory-specific regions undergo cross-modal plasticity, becoming responsive to non-auditory sensory modalities (Sandmann et al., 2012), including somatosensory stimuli. While Karns et al. (2012) utilized fMRI to examine hemodynamic responses associated with cortical activation, our study, using EEG source analysis, provides a higher temporal resolution perspective on how SSD influences the neural dynamics of somatosensory processing. Notably, we observed source activation at early latencies, specifically during the P50 CSEP component, indicating that cross-modal plasticity in individuals with SSD emerges at the initial stages of somatosensory cortical processing. The presence of early-latency exogenous P50-related source activity in auditory regions suggests that these areas are recruited for basic sensory encoding rather than higher-order cognitive processing. This contrasts with later-latency endogenous somatosensory responses (e.g., P3b), which are more reflective of attentional and decision-making processes. Our findings support the idea that Heschl’s gyrus is engaged in the earliest stages of somatosensory perception in individuals with acquired SSD, likely caused by functional reallocation following auditory deprivation. Together, these results reinforce the notion of auditory cortex playing an important role in early-stage exogenous somatosensory encoding, underscoring the adaptability of sensory processing networks in response to hearing loss.

Further supporting our results, Auer et al. (2007) demonstrated that vibrotactile stimulation elicits activation in the auditory cortices of individuals with bilateral postlingual deafness, providing additional evidence of cross-modal engagement of the auditory system in response to somatosensory input. Their fMRI findings suggest that the auditory cortex in bilateral postlingual deafness is not dormant but instead becomes functionally responsive to tactile stimulation, reinforcing the notion that early cortical recruitment for somatosensory processing may be a key compensatory mechanism. Our study expands on these findings by showing that such cross-modal plasticity is already evident at early exogenous sensory processing stages, as reflected by P50 activation patterns in individuals with acquired SSD. Similarly, Cardon and Sharma (2018), using EEG to investigate somatosensory cross-modal reorganization in adults with bilateral ARHL reported that individuals with mild to moderate ARHL demonstrated activation of auditory cortical regions in response to somatosensory stimulation. This cross-modal reorganization was also associated with decreased speech perception in noise, indicating functional implications of such neural changes (Cardon and Sharma, 2018).

In summary, our findings provide preliminary evidence of somatosensory cross-modal plasticity in acquired SSD, and reinforce the notion of the auditory cortex playing a role in early-stage somatosensory encoding. Our results also highlight the adaptability of sensory processing networks in response to auditory deprivation. The convergence of evidence from EEG and fMRI studies suggests that cortical reorganization in SSD extends beyond later cognitive processing stages to influence the fundamental neural encoding of somatosensory stimuli at early peak times. The distinct neural adaptations in LSSD and RSSD highlight differential compensatory mechanisms following auditory deprivation and suggest potential functional implications for sensory integration in SSD.

There are limitations to our study that should be taken into account when interpreting the results. One is the lower statistical power of some tests, which may have hindered the ability to detect subtle effects between the groups and subgroups comparisons. Additionally, the results from the present study are based on a heterogeneous study cohort in respect to onset of hearing impairment (peri- and postlingual), etiology, duration of deafness and age at onset of deafness. Moreover, somatosensory stimulation was applied only to the left index finger, which limits our understanding of cortical plasticity that involves both hemispheres. While the number of electrodes used here gave us valuable insights about the differences in the underlying source activation, a more localized analysis of the brain activation would need a larger number of electrodes (~256 electrodes) to avoid mislocalizations and blurring effects (Song et al., 2015). Further investigations using a longitudinal study design, larger sample sizes, bilateral stimulation design and higher electrode number, will be necessary to confirm and extend these findings. Nevertheless, understanding these changes prior to cochlear implantation might allow us to establish preoperative biomarkers for predicting CI outcome in adults with acquired SSD more precisely. Such biomarkers should optimize the indication and therapy and potentially facilitate the development of personalized auditory rehabilitation strategies following treatment with CI.

## Data Availability

The raw data supporting the conclusions of this article will be made available by the authors, without undue reservation.
