# Supplementary material for: Somatosensory cross-modal activation and changes in cortical somatosensory evoked potential responses in single-sided deafness: an EEG study
Source: Front Neurosci. 2025 Sep 5;19:1618134. doi: 10.3389/fnins.2025.1618134 (PMC12446350; doi:10.3389/fnins.2025.1618134)
Supplement: Supplementary file 1 [file Data_Sheet_1.docx]

Supplementary Material

# Supplementary table 1. Characteristics of included participants with SSD

| **ID** | **Age (years)** | **Sex** | **Hearing-impaired ear** | **Etiology of SSD** | **Duration of deafness (years)** | **Age at deafness onset** | **Air-conduction PTA4 (dB HL)** | |
| --- | --- | --- | --- | --- | --- | --- | --- | --- |
|  |  |  |  |  |  |  | **Better-hearing ear** | **Poorer hearing ear** |
| SSD 1 | 35.8 | male | right | unknown | 28.8 | 7.0 | 0.75 | 70.75 |
| SSD 2 | 55.1 | male | right | intracochlear schwannoma | 6.6 | 48.5 | 5.25 | 97.75 |
| SSD 3 | 50.9 | female | left | mastoiditis | 0.4 | 50.5 | 4.50 | 115.00 |
| SSD 4 | 63.8 | female | right | sudden hearing loss | 0.5 | 63.3 | 2.75 | 130.00 |
| SSD 5 | 32.4 | male | left | sudden hearing loss | 0.4 | 31.9 | 8.50 | 113.75 |
| SSD 6 | 58.8 | female | left | endolymphhydrops | 5.9 | 52.9 | 4.50 | 115.00 |
| SSD 7 | 44.6 | male | right | sudden hearing loss | 3.2 | 41.4 | 13.50 | 130.00 |
| SSD 8 | 38.6 | male | left | head trauma | 27.0 | 11.4 | 10.75 | 70.00 |
| SSD 9 | 47.1 | male | left | head trauma | 1.3 | 45.8 | 4.50 | 115.00 |
| SSD 10 | 50.6 | male | right | sudden hearing loss | 31.8 | 18.8 | 35.00 | 130.00 |
| SSD 11 | 36.8 | female | right | sudden hearing loss | 0.7 | 36.1 | 8.50 | 99.25 |
| SSD 12 | 51.8 | male | right | sudden hearing loss | 1.1 | 50.7 | 16.00 | 101.25 |
| SSD 13 | 52.6 | male | right | sudden hearing loss | 1.0 | 51.6 | 8.75 | 86.00 |
| SSD 14 | 57.8 | male | left | sudden hearing loss | 1.7 | 56.0 | 2.00 | 81.75 |
| SSD 15 | 57.8 | male | left | sudden hearing loss | 1.1 | 56.7 | 7.50 | 78.75 |
| SSD 16 | 66.1 | female | right | intracochlear schwannoma | 9.8 | 56.2 | 3.75 | 102.50 |
| SSD 17 | 47.1 | male | right | head trauma | 40.8 | 6.3 | 5.25 | 71.75 |
| SSD 18 | 36.8 | female | right | sudden hearing loss | 0.7 | 36.1 | 13.25 | 108.75 |
| SSD 19 | 65.6 | female | left | sudden hearing loss | 4.9 | 60.8 | 20.50 | 101.25 |
| SSD 20 | 58.5 | female | left | vestibularis schwannoma | 0.2 | 58.2 | 7.00 | 130.00 |

Supplementary table 2: Behavioral performance measures for the Left Single-Sided deaf (LSSD) and Right Single-Sided Deaf (RSSD) groups.

|  | LSSD (n = 9) | RSSD (n = 11) |  |
| --- | --- | --- | --- |
|  | Median (Min – Max) | Median (Min – Max) | p-value |
| Hit rates | 70 (57 – 78) | 70 (53 – 76) | 0.731 |
| False alarms | 4 (2 – 8) | 4 (1 – 11) | 1.000 |
| Misses | 10 (1 – 23) | 10 (4 – 27) | 0.731 |
| Correct rejections | 316 (312 – 318) | 316 (308 – 318) | 1.000 |
| Accuracy (%) | 88 (71 – 98) | 87 (66 – 96) | 0.731 |
| d' | 3.50 (2.61 – 4.63) | 3.70 (2.11 – 4.12) | 0.474 |
| log β | 1.64 (1.29 – 1.86) | 1.50 (0.81 – 1.73) | 0.964 |
| Hit RT (ms) | 458.50 (422.13 – 554.0) | 499.87 (468.22 – 541.57) | 0.835 |

*Significance level of 0.05* and of 0.01**. RT is for reaction time. SD is for standard deviation. Hit rates, false alarms, misses, correct rejections are reported as counts. d' and log β are dimensionless statistical measures.

**Supplementary Table 3:** Correlations between ERP component amplitudes and participant characteristics (n = 20, SSD group). Spearman’s rank-order or Pearson’s correlation was used depending on normality assumptions.

| **ERP Component** | **Variable*** | **Correlation coef.** | **p-value** |
| --- | --- | --- | --- |
| **P50** | Age | 0.190 | 0.410 |
|  | Duration of deafness | 0.122 | 0.598 |
|  | PTA4 right ear | -0.102 | 0.660 |
|  | PTA4 left ear | 0.133 | 0.565 |
| **N70** | Age | -0.111 | 0.631 |
|  | Duration of deafness | 0.467 | 0.033 |
|  | PTA4 right ear | 0.051 | 0.827 |
|  | PTA4 left ear | 0.227 | 0.321 |
| **P100** | Age | 0.222 | 0.334 |
|  | Duration of deafness | -0.142 | 0.538 |
|  | PTA4 right ear | -0.180 | 0.435 |
|  | PTA4 left ear | 0.402 | 0.071 |
| **N140** | Age | -0.054 | 0.816 |
|  | Duration of deafness | 0.119 | 0.608 |
|  | PTA4 right ear | -0.224 | 0.328 |
|  | PTA4 left ear | 0.487 | 0.025 |
| **P3b** | Age | -0.094 | 0.684 |
|  | Duration of deafness | -0.322 | 0.155 |
|  | PTA4 right ear | -0.247 | 0.280 |
|  | PTA4 left ear | 0.055 | 0.812 |

***** PTA = Air conduction Pure-Tone Average (in dB HL); Age and duration of deafness is in years.

## Supplementary figure 1:


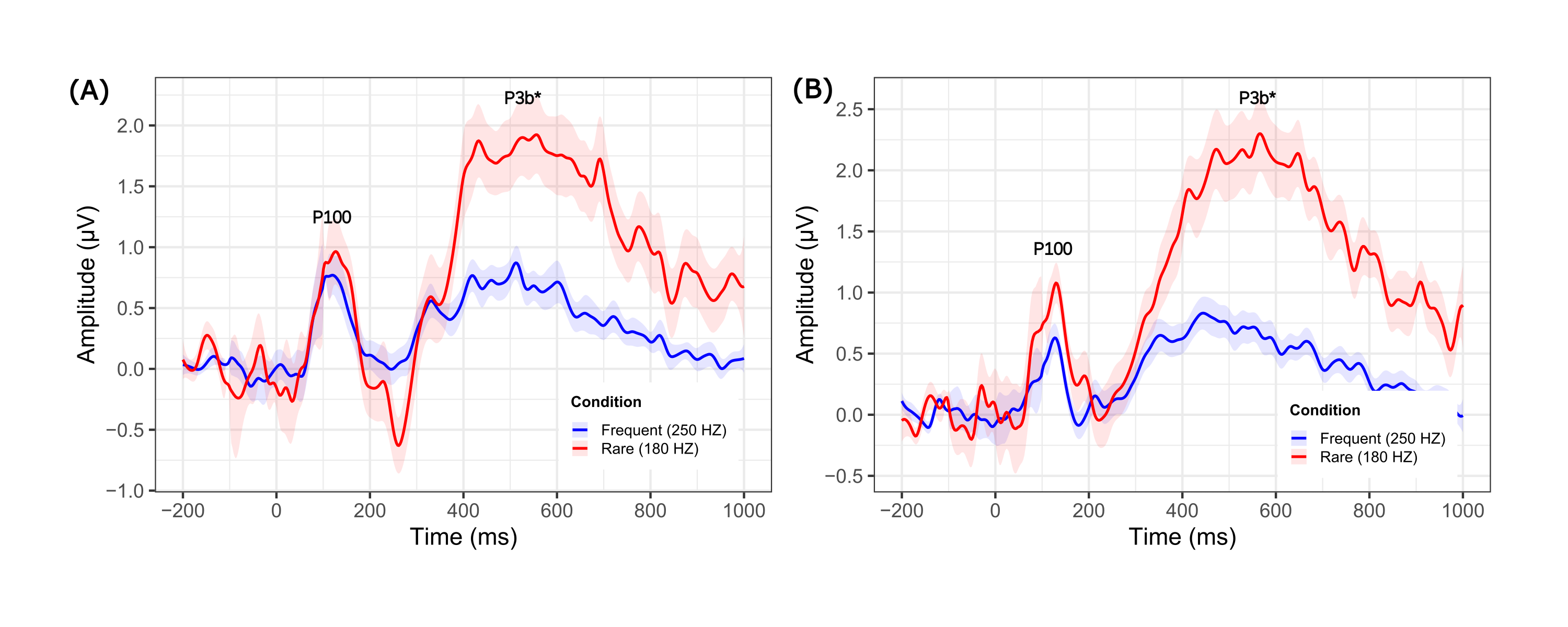


*Figure 1. Grand average cortical somatosensory evoked potentials (CSEPs) responses to frequent (250 Hz) and rare (180 Hz) vibrotactile stimuli in the NH (panel A) and SSD (panel B) groups at the right parietal (Rpar) region of interest. The solid red line represents the response to frequent stimuli while the solid blue line represents the response to rare stimuli. Shaded areas around the waveforms indicate standard error of the mean. Key CSEPs components (P50, N70, P100, N140, and P3b) are labeled at their peak times. Significantly different CSEP peak amplitudes are denoted by asterisks—single asterisks indicate significance at the p = 0.05 level.*

## Supplementary figure 2:


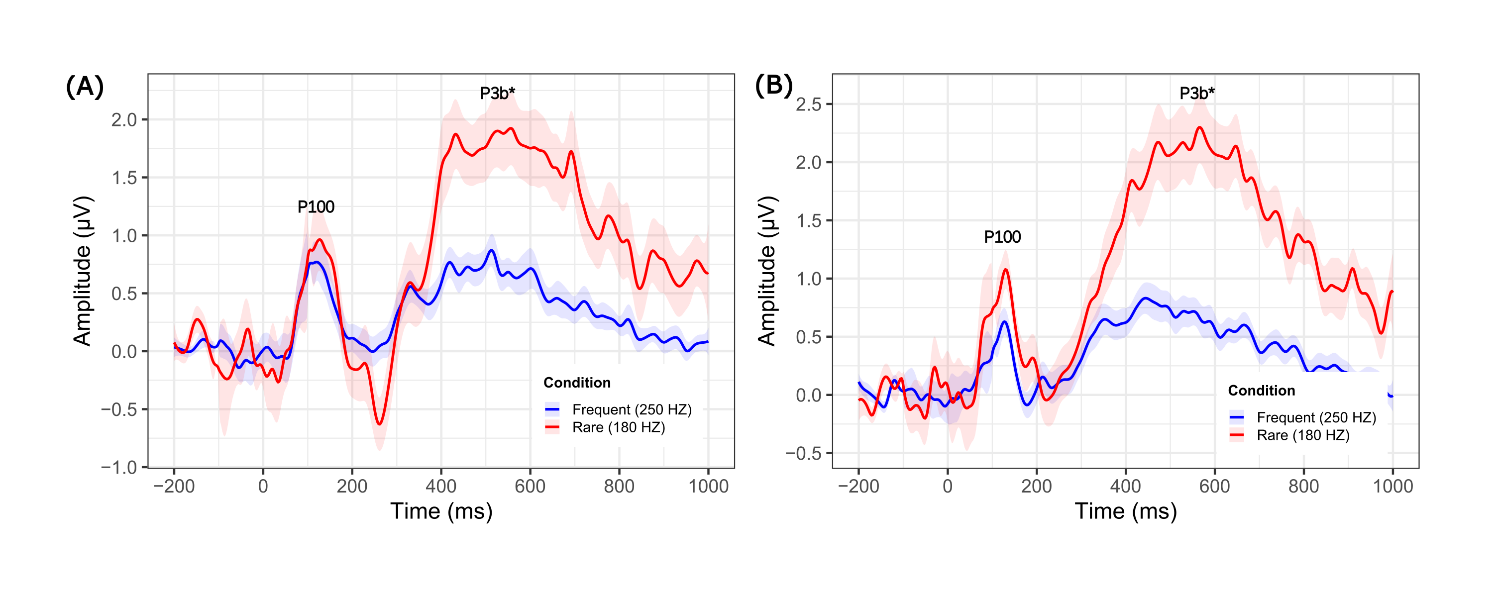


*Figure 2. Grand average cortical somatosensory evoked potentials (CSEPs) and topographical scalp distributions to the frequent tactile stimulus. The left panel (A) compares CSEP responses between NH (blue solid line) and SSD (dashed violet line) at the right parietal (Rpar) region of interest (ROI). The right panel (B) compares the CSEP responses between subjects individuals with left single-sided deafness (LSSD, n =9, blue solid line) and right single-sided deafness (RSSD, n = 11, red dashed line) for the right parietal ROI. The shaded areas represent the standard error of the mean. The key somatosensory evoked potential (CSEP) components—P50, N70, P100, and N140—are labeled.*

## Supplementary figure 3:


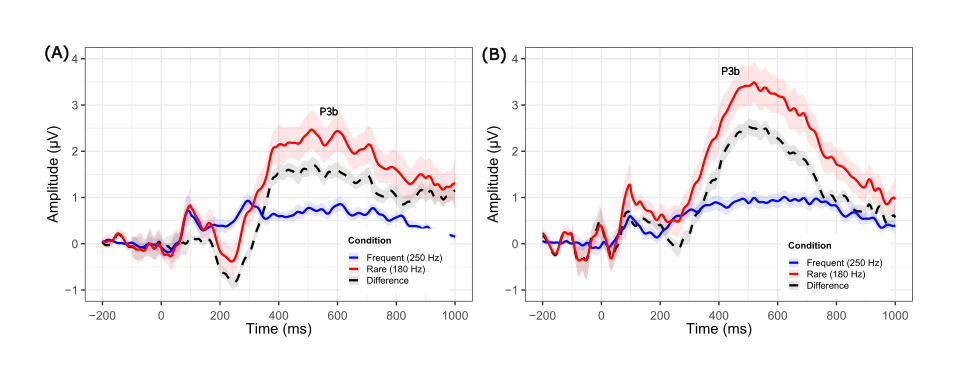


*Figure 3. Grand average cortical somatosensory evoked potentials (CSEPs) responses to frequent (250 Hz) and rare (180 Hz) vibrotactile stimuli, including difference waveform in the NH (panel A) and SSD (panel B) groups at the central region of interest. The solid red line represents the response to frequent stimuli while the solid blue line represents the response to rare stimuli. The dashed line represents the difference waveform obtained by substracting the frequent waveform amplitudes from the rare. Shaded areas around the waveforms indicate standard error of the mean.*

## Supplementary figure 4:


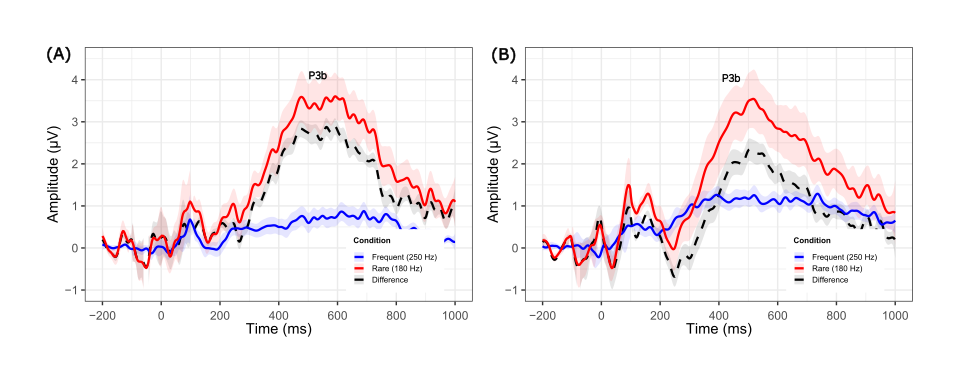


*Figure 4. Grand average cortical somatosensory evoked potentials (CSEPs) responses to frequent (250 Hz) and rare (180 Hz) vibrotactile stimuli, including difference waveform in the LSSD (panel A) and RSSD (panel B) groups at the central region of interest. The solid red line represents the response to frequent stimuli while the solid blue line represents the response to rare stimuli. The dashed line represents the difference waveform obtained by substracting the frequent waveform amplitudes from the rare. Shaded areas around the waveforms indicate standard error of the mean.*
